# Comment on “*Toxoplasma gondii* Infection in Pregnant Women: A Seroprevalence and Case-Control Study in Eastern China”

**DOI:** 10.1155/2016/5412168

**Published:** 2016-09-14

**Authors:** Siddharudha Shivalli, Soujanya Kaup

**Affiliations:** ^1^Department of Community Medicine, Yenepoya Medical College, Yenepoya University, Mangalore, Karnataka 575018, India; ^2^Department of Ophthalmology, Yenepoya Medical College, Yenepoya University, Mangalore, Karnataka 575018, India

Cross-sectional analytical and case-control studies are commonly reported study designs in clinical and public health research. Clinicians and researchers often get confused between them. Both are analytical observational studies, compare two study groups, and collect the data at one time from the study participants. However, the point of initiation of the study and interpretation of the results are quite different.

We came across an interesting article titled “*Toxoplasma gondii* Infection in Pregnant Women: A Seroprevalence and Case-Control Study in Eastern China” by Cong et al. [1]. According to the authors, this case-control study was performed to estimate the seroprevalence of* T. gondii* infection in pregnant women in two regions of eastern China and to identify associated risk factors and possible routes of contamination. However, looking carefully at the study method and reported results, this is a cross-sectional analytical study and not a case-control study as claimed by the authors. Seroprevalence cannot be estimated in a case-control study as the denominator (population at risk) is not available. To explore the risk factors of* T. gondii* infection in pregnant women through a case-control study, authors should have selected cases with the outcome (pregnant women with* T. gondii* infection) and controls (pregnant women without* T. gondii* infection).

Authors have studied large number of study participants (*n* = 1930); however, they should have justified the sample size. Assuming a seroprevalence of 9% among pregnant women in the reference population and after applying continuity correction, the study would require a sample size of 675 for each group (i.e., a total sample size of 1930, assuming equal group sizes), to achieve a power of 80% for detecting a difference in proportions of 5% between the test and the reference group at a two-sided *P* value of 0.05 [2].

The objective of the study was to identify the risk factors of* T. gondii* infection among pregnant women. However, the independent correlation between pregnancy and* T. gondii* infection is not analyzed by the authors. The applied regression model explains the independent correlates of* T. gondii* infection among all the study participants (pregnant and nonpregnant). In the result section, authors state that anti-*T. gondii* IgG antibodies were found in 147 (15.2%) of 965 pregnant women and in 167 (17.3%) of 965 control subjects (*P* = 0.217). The study variables were included in the multivariate analysis if they had a *P* value ≤ 0.25 in the bivariate analysis. In view of the study objective and *P* = 0.217 (i.e., less than 0.25) for pregnant versus control group, the pregnancy status should have been included in the regression analysis. In addition, it is always recommended to assess and report the adequacy of applied regression model. Failure to do so may lead to misleading or incorrect inferences. Although the study sample was very large (*n* = 1930), a word about *R* ^2^ (explaining the variance in outcome variable) of the applied regression model would have been more affirmative.

Nonetheless, we must congratulate the authors for investigating an important public health problem.
